# Changes in emergency department utilisation in Germany before and during different phases of the COVID-19 pandemic, using data from a national surveillance system up to June 2021

**DOI:** 10.1186/s12889-023-15375-7

**Published:** 2023-05-02

**Authors:** Madlen Schranz, T. Sonia Boender, Timo Greiner, Theresa Kocher, Birte Wagner, Felix Greiner, Jonas Bienzeisler, Michaela Diercke, Linus Grabenhenrich, AKTIN-Research Group, Jörg Brokmann, Carsten Mach, Markus Wehler, Sabine Blaschke, Katrin Esslinger, Domagoj Schunk, Sebastian Wolfrum, Tobias Hofmann, Benjamin Lucas, Matthias Klein, Thomas Peschel, Caroline Grupp, Hardy Wenderoth, Oliver Horn, Christian Wrede, Heike Hoeger-Schmidt, Harald Dormann, Greta Ullrich, Kirsten Habbinga, Thomas Henke, Tobias Schilling, Bernadett Erdmann, Eckart Wetzel, Markus Baacke, Rupert Grashey, Rainer Röhrig, Raphael Majeed, Alexander Kombeiz, Lucas Triefenbach, Felix Walcher, Wiebke Schirrmeister, Ronny Otto, Susanne Drynda, Annette Aigner, Alexander Ullrich

**Affiliations:** 1 Department for Infectious Disease Epidemiology, Robert Koch Institute, Berlin, Germany; 2 Charité – Universitätsmedizin Berlin, corporate member of Freie Universität Berlin and Humboldt, Universität zu Berlin, Institute of Public Health, Berlin, Germany; 3 Department for Methods Development, Research Infrastructure and Information Technology, Robert Koch Institute, Berlin, Germany; 4 Department of Trauma Surgery, Otto von Guericke University Magdeburg, Magdeburg, Germany; 5 Institute for Occupational and Maritime Medicine (ZfAM), University Medical Center Hamburg-Eppendorf (UKE), Hamburg, Germany; 6 Institute of Medical Informatics, Medical Faculty, RWTH Aachen University, Aachen, Germany; 7 Charité – Universitätsmedizin Berlin, Institute of Biometry and Clinical Epidemiology, Berlin, Germany

**Keywords:** Syndromic surveillance, Emergency department, COVID-19 pandemic, Routine data, Interrupted time series analyses, Public health and social measures

## Abstract

**Background:**

During the COVID-19 pandemic and associated public health and social measures, decreasing patient numbers have been described in various healthcare settings in Germany, including emergency care. This could be explained by changes in disease burden, e.g. due to contact restrictions, but could also be a result of changes in utilisation behaviour of the population. To better understand those dynamics, we analysed routine data from emergency departments to quantify changes in consultation numbers, age distribution, disease acuity and day and hour of the day during different phases of the COVID-19 pandemic.

**Methods:**

We used interrupted time series analyses to estimate relative changes for consultation numbers of 20 emergency departments spread throughout Germany. For the pandemic period (16-03-2020 – 13-06-2021) four different phases of the COVID-19 pandemic were defined as interruption points, the pre-pandemic period (06-03-2017 – 09-03-2020) was used as the reference.

**Results:**

The most pronounced decreases were visible in the first and second wave of the pandemic, with changes of − 30.0% (95%CI: − 32.2%; − 27.7%) and − 25.7% (95%CI: − 27.4%; − 23.9%) for overall consultations, respectively. The decrease was even stronger for the age group of 0–19 years, with − 39.4% in the first and − 35.0% in the second wave. Regarding acuity levels, consultations assessed as urgent, standard, and non-urgent showed the largest decrease, while the most severe cases showed the smallest decrease.

**Conclusions:**

The number of emergency department consultations decreased rapidly during the COVID-19 pandemic, without extensive variation in the distribution of patient characteristics. Smallest changes were observed for the most severe consultations and older age groups, which is especially reassuring regarding concerns of possible long-term complications due to patients avoiding urgent emergency care during the pandemic.

**Supplementary Information:**

The online version contains supplementary material available at 10.1186/s12889-023-15375-7.

## Background

The COVID-19 pandemic and associated public health and social measures have influenced population health and various aspects of healthcare in Germany. Following the first introduction of nation-wide public health measures in 2020, emergency department consultations in Germany decreased by about 40% [1, 2]. Similar trends were observed in other countries [3–5]. Syndromic surveillance can contribute to the understanding of this rather indirect impact of the COVID-19 pandemic and associated measures on public health, by monitoring the population’s healthcare seeking behaviour [6]. This monitoring can be based on the analysis of routinely collected data from emergency departments (EDs), without creating additional burden for the staff on site. The Robert Koch Institute (RKI) has been developing an emergency department surveillance system using data from about 35 emergency departments spread over 11 federal states in Germany. In reaction to the COVID-19 pandemic and its possible influences on healthcare seeking behaviour, and to inform the RKI and other German public health authorities, a situation report has been implemented starting from June 2020, displaying the overall emergency department consultations, stratified by age group, disease acuity, and certain presenting complaints [2]. The report is published on the RKI website on a weekly basis [7] and has been part of RKI’s COVID-19 situation reports [8]. The report gives a descriptive overview of the general situation in participating emergency departments. However, a deeper understanding of the utilisation behaviour can only be achieved by further analyses of patient dynamics during the pandemic.

Observed changes in the number of emergency department consultations might have been due to actual changes in disease burden, e.g. with fewer consultations for traumas (injuries, accidents) due to reduced mobility and cancellation of mass gatherings [1]. On the other hand, they could have been the result of changes in utilisation behaviour, as e.g. patients avoided seeking healthcare being concerned about getting infected with COVID-19 [9, 10]. Especially with the reduced consultations for acute events like myocardial infarction and stroke, concerns arose that patients would forego essential emergency treatment or lack the access to healthcare due to lockdown measures. In turn, this could lead to a later “rebound” effect of long-term complications and a higher number of deaths outside the healthcare system [1, 6, 11] and could call for increased risk communication and public health messaging, to raise awareness of the importance of emergency care, even in pandemic situations. Decreasing patient and case numbers have been observed in different settings of acute care, across all age groups, levels of acuity, and chief complaints. Among the most severe diagnoses, especially the number of patients presenting with cardiovascular emergencies and stroke have decreased during the first wave of the COVID-19 pandemic [1, 12, 13].

Most studies conducted in Germany focused on specific health outcomes, analysed only changes occurring during the first wave of the pandemic, or included data from single study sites. The present study goes beyond these limitations. To give a more comprehensive picture of patient characteristics during the COVID-19 pandemic, and to be able to inform a timely surveillance of changes in ED utilisation and patient characteristics, it includes observations form a national surveillance system including emergency departments across Germany, spanning across 15 months and 4 pandemic phases, and compares them to a 3-year pre-pandemic reference period. Based on this data, this study aims to assess trends in the number of overall emergency department consultations, but also their age distribution and acuity, just as the timing of consultation (including day of the week and time of day).

## Methods

### Study setting and variables

We conducted a time series analyses based on routine data from emergency departments, which either participated in the ESEG project [14] or are part of the AKTIN emergency department data registry [15, 16], whose data are sent to RKI in daily data exports. For both projects, inclusion of the EDs is pragmatically based on voluntary participation and not on representativity for all EDs in Germany. Each observation represents an electronic health record of a single emergency department consultation, multiple consultations of a specific patient cannot be identified in our dataset. The patient-level data is anonymized and pre-processed according to a generic data model (NotaufnahmeKernDatensatz, NoKeDa), which ensures usability of the data across different ED information systems and reporting standards [17]. For the present study, the following variables were included for analysis: age in groups of 0–19 years, 20–39 years, 40–59 years, 60–79 years, 80+ years (originally collected as: 0–4 years in 1-year age groups, 5–79 in 5-year age groups, 80+ years), acuity level (1 = immediate, 2 = very urgent, 3 = urgent, 4 = standard, 5 = non urgent) according to primary assessment stemming either from the Manchester Triage System [18] or the Emergency Severity Index [19], as well as weekday and hour of each consultation. The choice of variables into the present study was mainly based on the variables used in our weekly surveillance report [7]. The variables age and acuity level are characterised by being relatively standardised across all emergency departments, by being operationalised clearly, by having virtually no missing values, and are the main descriptors of patient characteristics in emergency departments. Additionally, weekday and hour of consultation are necessary to explore, whether the typical time pattern of emergency department utilisation has changed during the pandemic.

### Study population

As reference data we included data on 3 years prior to the pandemic starting with 06-03-2017. Data for the pandemic could be included until 13-06-2021 which marked the end of the ESEG project. We included data from emergency departments which provided at least one consultation recorded for each day of the study period. Every consultation recorded within this period fulfilling a minimal requirement for data transfer was included, i.e. information on age, day and hour of consultation provided.

### Statistical analyses

Descriptive analyses included the visualisation of the overall number of consultations over time, as well as their distribution of age, acuity levels, and consultation timing.

To quantify the changes in patient dynamics during the COVID-19 pandemic, we conducted interrupted time series analyses using negative binomial regression (Eq. 1)

1 $$\log (count)={\textrm{\ss}}_0+{\sum}_{i=1-4}{\textrm{\ss}}_i\ast {pandemic\ phase}_i$$

Equation 1 – negative binomial regression model used to estimate relative change in number of consultations

From the exponential of the regression coefficients ß_i_ for the pandemic phase indicator variables we derived the relative differences between the reference pre-pandemic phase and the respective pandemic phases. The interruptions over time were defined by the pandemic phases, as proposed by Tolksdorf et al. [20]:

1. First COVID-19 wave between weeks 10–2020 and 20–2020
2. Summer plateau between weeks 21–2020 and 39–2020
3. Second COVID-19 wave between weeks 40–2020 and 08–2021
4. Third COVID-19 wave between weeks 09–2021 and 23–2021

The dependent variable was the number of overall weekly consultations, the interruptions (independent variable) were defined as the pre-pandemic period (start of study period until start of wave 1) and the respective pandemic phases. For the categorical variables age, acuity level, weekday and hour of consultation, stratified regression analyses were performed for each value of the respective variable. Using the pre-pandemic period as the reference category in all regression models, we herewith obtain the relative change for each category, irrespective of trends or changes in the other categories.

To account for the potentially not immediate change in emergency department utilisation at the start of the pandemic phases, we investigated the model fit of three different models with the Akaike Information Criterion (AIC): fitting the interruption with A) a two-week delay, B) a one-week, and C) no delay from the starting point of each phase. The model using a two-week delay period from the start of each pandemic phase as interruption points showed the best fit (Supplementary Fig. 1). We additionally modelled all analyses including time as a linear variable up to the first interruption, and looked at its effect size and statistical significance to decide, whether or not to include time in the final models. Only small changes (between 0.04% and − 0.01%) could be observed across the stratified analyses (Supplementary Table 1), indicating that consultation numbers did not show major time trends before the start of the COVID-19 pandemic. We therefore decided not to include time in the final model.

All analyses were performed using R version 4.1.2 [21] and the packages tidyverse [22], MASS [23], finalfit [24], xlsx [25], Table 1 [26], and ggpubr [27].

**Table 1:** Relative change in % and 95 confidence interval for all emergency department consultations, consultations by age group, acuity level, weekday, and hour of day, comparing every pandemic phase with the pre-pandemic reference period

	*% change [95% CI]*				
	Pre-Pandemic	Wave 1	Summerbreak	Wave 2	Wave 3
**All attendances**					
	Reference	−29.98% [− 32.21%; − 27.69%]	−8.30% [− 10.57%; − 5.97%]	− 25.66% [− 27.38%; − 23.89%]	−14.49% [− 17.00%; − 11.91%]
**Age group**					
0–19	Reference	− 39.37% [− 43.83%; − 34.56%]	− 7.74% [− 13.04%; − 2.13%]	−35.01% [− 38.53%; − 31.29%]	− 16.01% [− 21.71%; − 9.89%]
20–39	Reference	−32.86% [− 35.50%; − 30.10%]	−11.56% [− 14.26%; − 8.78%]	− 31.57% [− 33.55%; − 29.54%]	−18.95% [− 21.89%; − 15.90%]
40–59	Reference	−26.56% [− 28.93%; − 24.11%]	−7.30% [− 9.60%; − 4.93%]	− 24.47% [− 26.25%; − 22.64%]	− 13.29% [− 15.86%; − 10.64%]
60–79	Reference	−23.95% [− 26.37%; − 21.45%]	−5.00% [− 7.33%; −2.60%]	− 18.72% [− 20.61%; − 16.79%]	−9.80% [− 12.43%; − 7.09%]
80+	Reference	−27.71% [− 30.15%; − 25.18%]	− 10.15% [− 12.49%; − 7.75%]	−17.43% [− 19.46%; − 15.35%]	−14.39% [− 17.04%; − 11.66%]
**Severity level**					
1 (immediate)	Reference	−24.30% [− 29.39%; − 18.83%]	−3.82% [− 8.60%; 1.20%]	−11.05% [− 15.27%; − 6.62%]	−1.48% [− 7.23%; 4.63%]
2 (very urgent)	Reference	−25.02% [− 27.95%; − 21.98%]	−6.42% [− 9.22%; − 3.53%]	−13.52% [− 15.96%; − 11.00%]	−8.16% [− 11.42%; − 4.77%]
3 (urgent)	Reference	−26.54% [− 29.06%; − 23.94%]	−3.96% [− 6.51%; − 1.34%]	−18.52% [− 20.56%; − 16.43%]	−7.73% [− 10.63%; − 4.72%]
4 (standard)	Reference	−37.23% [− 40.06%; − 34.26%]	−11.98% [− 15.07%; − 8.77%]	− 35.49% [− 37.63%; − 33.28%]	−20.96% [− 24.26%; − 17.53%]
5 (non urgent)	Reference	−31.93% [− 37.69%; − 25.64%]	−3.17% [− 9.51%; 3.61%]	− 29.21% [− 33.62%; − 24.50%]	−17.86% [− 24.25%; − 10.92%]
Missing	Reference	− 8.28% [− 13.81%; − 2.40%]	− 18.60% [− 22.47%; − 14.53%]	− 27.06% [− 30.34%; − 23.63%]	− 25.75% [− 29.70%; − 21.59%]
**Weekday**					
Monday	Reference	−28.93% [− 31.77%; − 25.96%]	−6.47% [− 9.35%; − 3.50%]	−22.72% [− 24.97%; − 20.39%]	− 13.11% [− 16.29%; − 9.81%]
Tuesday	Reference	−28.27% [− 30.94%; − 25.50%]	−5.85% [− 8.53%; − 3.09%]	− 22.25% [− 24.35%; − 20.08%]	− 12.02% [− 15.01%; − 8.93%]
Wednesday	Reference	−28.79% [− 31.47%; − 26.00%]	− 7.56% [− 10.24%; − 4.81%]	−24.08% [− 26.16%; − 21.93%]	−12.92% [− 15.92%; − 9.82%]
Thursday	Reference	−28.88% [− 31.80%; − 25.84%]	− 8.24% [− 11.15%; − 5.24%]	−24.93% [− 27.18%; − 22.60%]	− 11.61% [− 14.93%; − 8.16%]
Friday	Reference	−29.93% [− 33.07%; − 26.65%]	−10.37% [− 13.47%; − 7.16%]	− 29.43% [− 31.74%; − 27.04%]	−16.96% [− 20.37%; − 13.41%]
Saturday	Reference	−34.04% [− 37.12%; − 30.82%]	− 11.90% [− 15.07%; − 8.61%]	−33.35% [− 35.63%; − 30.99%]	− 20.14% [− 23.56%; − 16.57%]
Sunday	Reference	−31.08% [− 33.85%; − 28.20%]	−7.83% [− 10.68%; − 4.90%]	−23.29% [− 25.53%; − 20.98%]	− 14.90% [− 18.02%; − 11.65%]
**Hour**					
0	Reference	− 32.26% [− 36.70%; − 27.50%]	−11.00% [− 15.38%; − 6.40%]	−34.45% [− 37.62%; − 31.11%]	−26.99% [− 31.38%; − 22.33%]
1	Reference	− 33.65% [− 38.47%; − 28.45%]	− 12.69% [− 17.44%; − 7.67%]	− 33.59% [− 37.14%; − 29.85%]	−26.90% [− 31.74%; − 21.71%]
2	Reference	−33.53% [− 38.26%; − 28.43%]	− 14.30% [− 18.83%; − 9.53%]	− 31.61% [− 35.16%; − 27.86%]	−27.48% [− 32.17%; − 22.46%]
3	Reference	−30.25% [− 35.20%; − 24.93%]	− 10.48% [− 15.16%; − 5.55%]	− 30.52% [− 34.14%; − 26.70%]	− 23.62% [− 28.52%; − 18.37%]
4	Reference	−27.22% [− 32.48%; − 21.55%]	−10.43% [− 15.24%; − 5.34%]	−26.16% [− 30.06%; − 22.04%]	−20.49% [− 25.70%; − 14.91%]
5	Reference	−19.90% [− 26.26%; − 12.98%]	5.07% [− 1.18%; 11.72%]	− 21.68% [− 26.28%; − 16.79%]	6.62% [− 0.85%; 14.65%]
6	Reference	−10.86% [− 18.10%; − 2.97%]	14.54% [7.44%; 22.10%]	− 9.93% [− 15.32%; − 4.20%]	45.17% [34.81%; 56.33%]
7	Reference	−16.13% [− 22.14%; − 9.65%]	14.03% [7.80%; 20.62%]	− 0.90% [− 6.04%; 4.53%]	30.57% [22.24%; 39.46%]
8	Reference	−27.39% [− 31.18%; − 23.39%]	−3.44% [− 7.26%; 0.54%]	− 16.53% [− 19.68%; − 13.26%]	−1.71% [− 6.31%; 3.12%]
9	Reference	−31.98% [− 34.65%; − 29.21%]	− 7.25% [− 9.96%; − 4.45%]	−21.52% [− 23.72%; − 19.25%]	− 11.04% [− 14.15%; − 7.83%]
10	Reference	−30.23% [− 33.02%; − 27.32%]	−10.63% [− 13.34%; − 7.84%]	− 20.76% [− 23.04%; − 18.41%]	− 14.24% [− 17.34%; − 11.03%]
11	Reference	− 31.52% [− 34.12%; − 28.83%]	−10.01% [− 12.57%; − 7.37%]	−20.78% [− 22.93%; − 18.57%]	−13.70% [− 16.62%; − 10.67%]
12	Reference	−28.67% [− 31.32%; − 25.92%]	−10.48% [− 12.98%; − 7.89%]	−21.66% [− 23.76%; − 19.51%]	− 14.21% [− 17.08%; − 11.24%]
13	Reference	−28.69% [− 31.40%; − 25.88%]	−7.85% [− 10.48%; − 5.15%]	−20.98% [− 23.14%; − 18.76%]	−15.79% [− 18.68%; − 12.81%]
14	Reference	−28.39% [− 31.20%; − 25.46%]	−7.03% [− 9.77%; − 4.20%]	−21.69% [− 23.91%; − 19.40%]	−14.19% [− 17.22%; − 11.04%]
15	Reference	−28.82% [− 31.53%; − 25.99%]	−8.38% [− 10.99%; − 5.68%]	−22.79% [− 24.92%; − 20.61%]	−12.34% [− 15.33%; − 9.25%]
16	Reference	−28.95% [− 31.55%; − 26.25%]	− 8.84% [− 11.33%; − 6.29%]	−22.34% [− 24.39%; − 20.25%]	−13.60% [− 16.42%; − 10.69%]
17	Reference	−29.57% [− 32.39%; − 26.64%]	−9.10% [− 11.83%; − 6.29%]	−25.73% [− 27.89%; − 23.52%]	−17.22% [− 20.20%; − 14.12%]
18	Reference	−31.73% [− 34.75%; − 28.57%]	− 10.16% [− 13.15%; − 7.06%]	−30.69% [− 32.93%; − 28.38%]	−17.47% [− 20.76%; − 14.04%]
19	Reference	−32.29% [− 35.35%; − 29.08%]	−9.22% [− 12.31%; − 6.02%]	−33.62% [− 35.83%; − 31.34%]	−17.99% [− 21.35%; − 14.50%]
20	Reference	−32.93% [− 36.52%; − 29.12%]	−8.95% [− 12.66%; − 5.08%]	−37.74% [− 40.21%; − 35.17%]	− 22.06% [− 25.87%; − 18.05%]
21	Reference	−31.90% [− 36.36%; − 27.12%]	−9.28% [− 13.84%; − 4.47%]	−40.02% [− 42.94%; − 36.94%]	−24.66% [− 29.20%; − 19.82%]
22	Reference	−34.68% [− 38.96%; − 30.09%]	− 12.86% [− 17.23%; − 8.27%]	−40.59% [− 43.48%; − 37.55%]	−27.66% [− 32.02%; − 23.02%]
23	Reference	−36.34% [− 40.69%; − 31.67%]	− 13.34% [− 17.81%; − 8.62%]	−39.93% [− 42.97%; − 36.74%]	− 27.75% [− 32.25%; − 22.95%]

## Results

Between 06-03-2017 and 13-06-2021, a total of 35 emergency departments transferred data from a total of 3,991,480 consultations to the RKI. Of these, 20 emergency departments fulfilled the inclusion criteria, leading to a final study sample of 3,143,273 consultations. The included emergency departments spread throughout 9 German federal states and cover all three levels of care according to Federal Joint Committee [28] (comprehensive care: 13 EDs, extended care: 5 EDs, basic care: 1 ED). Average consultation numbers per emergency department range from about 200 up to 1600 per week. For 5.6% of consultations (*n* = 176,747) primary assessment of acuity was missing, i.e. it was not carried out or not recorded.

### Overall consultations

**Fig. 1:**
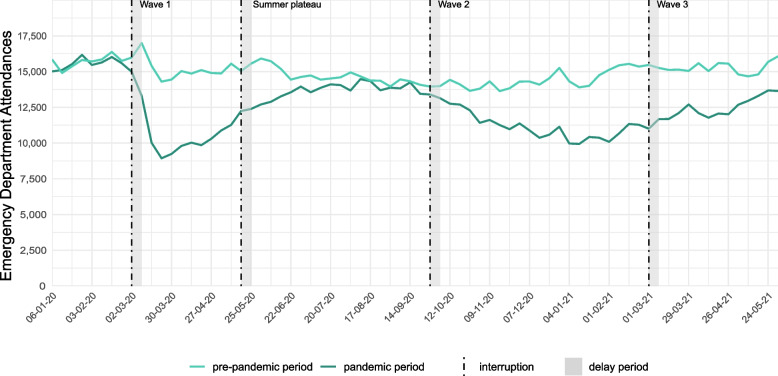
Absolute weekly number of emergency department consultations in the pre-pandemic period (01-01-2018 – 01-06-2019) compared to the pandemic period (01-01-2020 – 01-06-2021); the x-axis refers to the pandemic period

Compared to the pre-pandemic period, a 30.0% (95%CI: − 32.2%; − 27.7%) decrease in emergency department consultations was detected during the first wave. Consultation numbers increased again during the summer plateau, however not reaching those recorded the year before, staying at − 8.3% [95%CI: − 10.6%; − 6%] compared to the reference period. During the second pandemic wave, consultations dropped again, with a relative change of − 25.7% (95%CI: − 27.4%; − 23.9%) compared to the pre-pandemic period, followed by an increase during the third wave, with a relative change of − 14.5% [95%CI: − 17.0%; − 11.9%] compared to the pre-pandemic period (Fig. 1, Supplementary Table 2, Supplementary Fig. 4).

### Age distribution

**Fig. 2:**
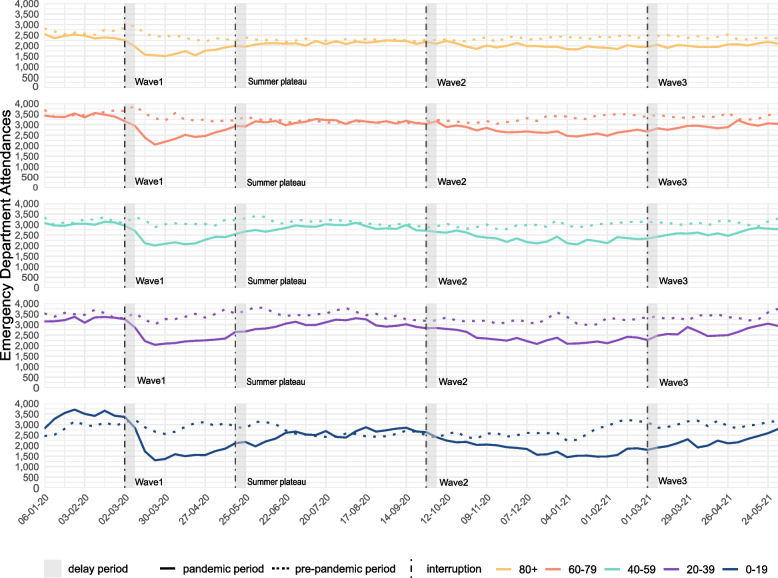
Absolute weekly number of emergency department consultations stratified by age group in the pre-pandemic period (01-01-2018 – 01-06-2019) compared to the pandemic period (01-01-2020 – 01-06-2021)

Before the beginning of the first wave of the COVID-19 pandemic (week 10–2020), 18.5% of all consultations were younger than 20 years and 16.0% over 80. Similar distributions were visible in the first and third wave. The number of consultations increased among the older age groups during the summer plateau, where the 0–19-year-olds made up between 16.5 and 17.2% of consultations while 60–79-year-olds increased to 23.4 and 24.0% (Fig. 2, Supplementary Table 2).

**Fig. 3:**
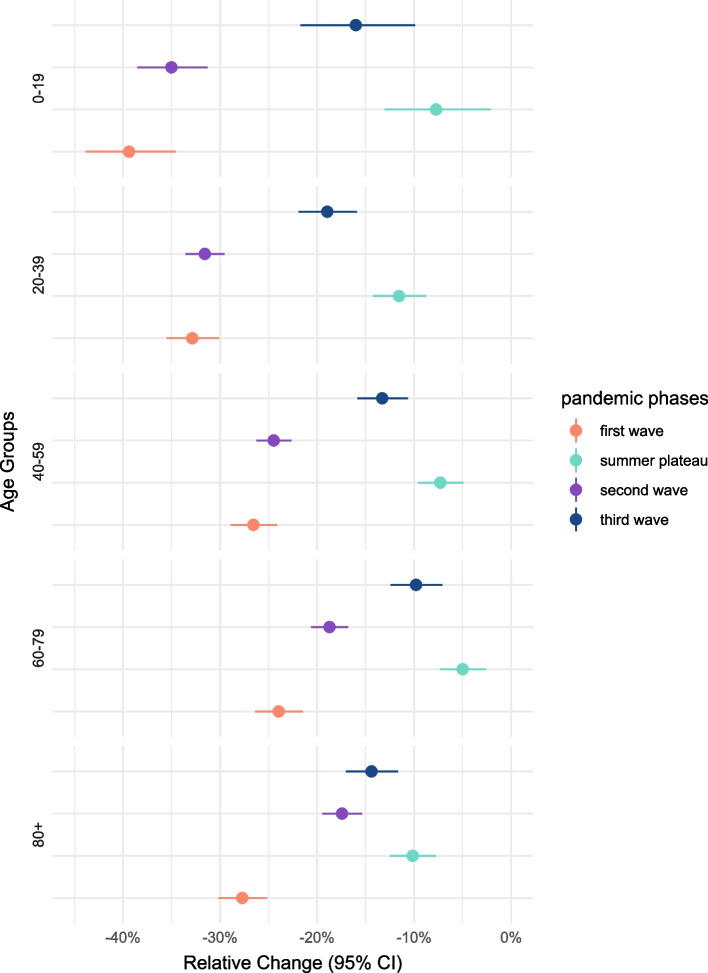
Relative change in % and 95 confidence interval for all emergency department consultations by age group, comparing every pandemic phase with the pre-pandemic reference period

The relative changes in the number of consultations for each age group showed similar trends as compared to the overall consultations. The decrease was most pronounced among young people (0–19 years), especially during the first and second waves (− 39.4% and − 35.0%, respectively). The smallest decrease in consultations was seen among those aged 60–79, with decreases between − 24 and − 5% throughout (Table 1**,** Fig. 3).

### Acuity level

**Fig. 4:**
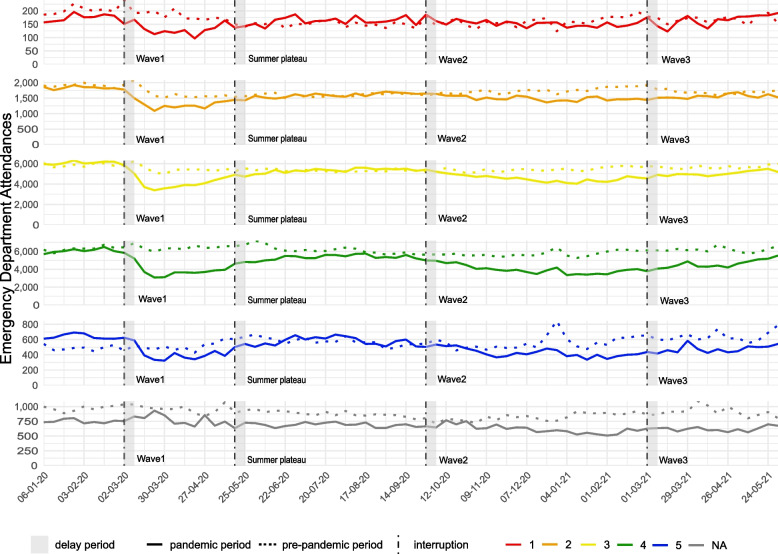
Absolute weekly number of emergency department consultations stratified by acuity level in the pre-pandemic period (01-01-2018 – 01-06-2019) compared to the pandemic period (01-01-2020 – 01-06-2021)

Before the pandemic, most consultations were assessed as 3-urgent (37.2%) or 4-standard (40.5%) level of acuity. Those two levels also had the highest numbers in all pandemic phases. The category with the fewest observations was 1-immediate, with 1.1 to 1.4% of consultations. Acuity levels 5-non-urgent accounted for around 4% of consultations. (Fig. 4, Supplementary Table 2).

**Fig. 5:**
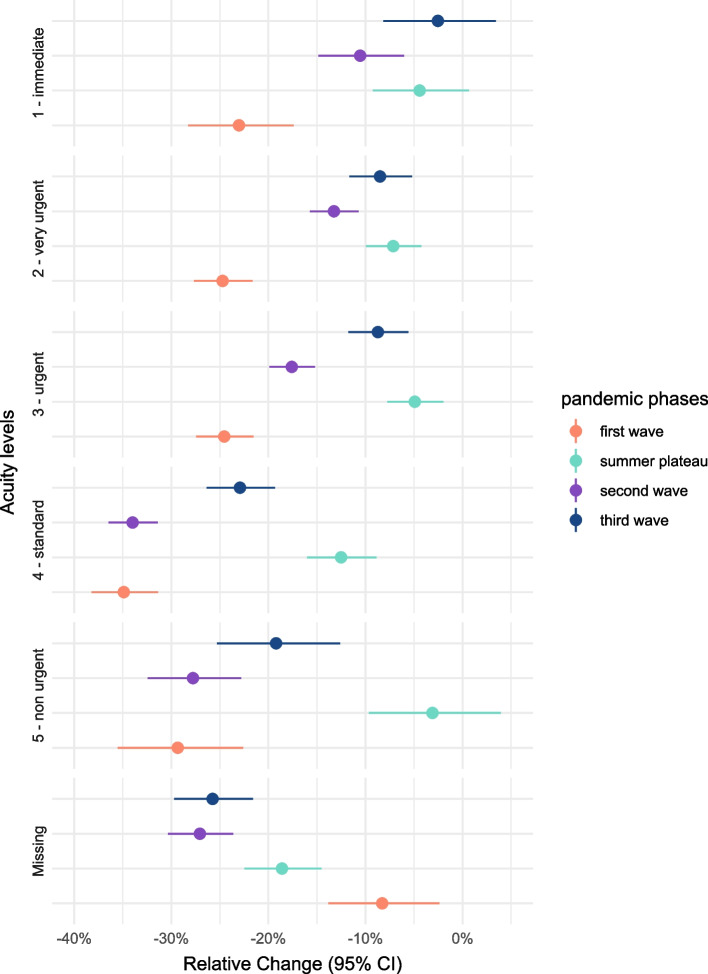
Relative change in % and 95 confidence interval for all emergency department consultations by acuity level, comparing every pandemic phase with the pre-pandemic reference period

A reduction of cases compared to the pre-pandemic time was especially visible for the acuity levels 3-urgent, 4-standard and 5-non-urgent, during the first wave of COVID-19. The most severe cases (acuity level 1) reduced by − 24.3% [95%CI: − 29.4%; − 18.8%] in the first and − 11.1% [95%CI: − 15.3%; − 6.6%] in the second wave, but were at a similar level as in the reference period during the summer plateau (− 3.8% [95%CI: − 8.6%; 1.2%]) and the third wave (− 1.5% [95%CI: − 7.2%; 4.6%]) (Table 1**,** Fig. 5).

### Weekday and hour of consultation

While consultations on Fridays and Saturdays were slightly fewer during the summer plateau, they were slightly increased for Tuesdays and Wednesdays during the second and third wave. Overall however, consultations were evenly distributed with 12.3 to 15.8% across all days of the week prior to the COVID-19 pandemic and in each pandemic phase. Prior to the pandemic, just as within all pandemic phases, most consultations occurred between 9 am and 1 pm, the fewest patients were recorded between 3 am and 5 am (Supplementary Table 2).

Based on the results from the interrupted time series analyses, consultations decreased by about − 30% for all days of the week in the first and second waves of the pandemic. Also, for the time of consultation, a reduction of about − 30% on average was recorded during the first COVID-19 wave (Table 1, Supplementary Fig. 5). As the only exception, the numbers dropped by − 10.9 and − 16.1% at 6 am and 7 am, while in the second and third wave of the pandemic the number of consultations increased during those hours up to 14.5 and 14.0% (second wave) and up to 45.2 and 30.6% (third wave), compared to pre-pandemic times (Supplementary Fig. 6).

## Discussion

### Key results

The COVID-19 pandemic and the associated public health and social measures have impacted emergency department utilisation in Germany. During the first wave of the COVID-19 pandemic a substantial and rapid decrease of emergency department consultations was observed, this change presented across all age groups and all acuity levels, weekdays, and hours of consultation. Similar trends were observed in the second wave. However, the decrease was much smaller during the summer plateau and the third wave. The drastic decrease of consultations at the start of the public health and social measures in Germany beginning in week 10–2020 is in accordance with studies and reports from other countries like the UK and the US [5, 29, 30]. In our analysis, the COVID-19 pandemic and associated measures had the most impact on moderately severe and non-urgent cases (acuity levels 3, 4, and 5). This might indicate that less urgent emergency department consultations were avoided. Similar findings were also described in a study from the UK, analysing the change in consultation counts during the first pandemic wave [4]. Generally, the number of most severe consultations were similar to the pre-pandemic phase, except for a decrease in the first wave, which is disconcerting and might further underpin the fear of unmet needs for acute events in the emergency department during the COVID-19 pandemic. The older age groups (60–79 years, 80+ years) showed the smallest decrease in the number of consultations, indicating that utilisation behaviour did not change within those more vulnerable groups and they were not lacking access due to public health and social measures. Generally speaking, the probability to be assessed with more urgent acuity levels and the necessity for an inpatient admission is increasing with increasing age. This also means that an immediate need for action is given, for many elderly patients there is no alternative to an ED consultation [31]. Meanwhile, the great and rapid decrease in the youngest age group could point towards parents not taking their children into emergency departments, possibly being concerned of an infection with SARS-CoV-2 [32]. In addition, the reduction of consultations in that age group might be related to a reduction of trauma-related injuries due to lockdown measures, suspension of sports activities and school closures [33]. Studies conducted in the US and UK during the first pandemic wave showed similar results, with the biggest reduction in consultations described for the age groups 0–19 [5], 5–14 [4], and children younger than 14 [30], and the smallest decrease in older age groups. The changes described for the timing of the consultations (i.e. weekday and time of day) are in accordance with the overall decrease in consultation numbers of about − 30%. This is contrary to findings from the UK, where the biggest reductions were reported for Monday through Wednesday [4]. This however may also be due to differences in COVID-19 measures, in the healthcare structure and access to emergency departments in these two countries.

### Strengths and limitations

The present study is using interrupted time series analyses to quantify the impact of public health and social measures associated with the COVID-19 pandemic. This gives additional insights into changes in emergency department utilisation as compared to descriptive analyses previously done [2]. We included 20 emergency departments across 9 states in Germany of different sizes and levels of care. Although we cannot assess the representativeness of the included emergency departments due to the lack of comparative data on the national level, we believe that this possible limitation is negligible in our study due to regional dispersion and the variables we have selected. As with all routinely collected secondary data, the quality of the data is dependent on documentation practice in the respective emergency department, which might for example lead to missing values in certain variables. While this can be challenging in many use cases of emergency department surveillance, it is assumed to be a minor problem for the present analyses. The only variable used in the present study that is affected by this is acuity level, where about 5% of all consultations either had no acuity level documented or were not assessed in triage as they had immediate physician contact. The nature of the data furthermore doesn’t allow us to identify structural changes within the emergency department. E. g. during the pandemic, single emergency departments opened special COVID-wards, where suspected cases were directly transferred. We don’t expect a large number of ED consultations to have been documented outside of the ED, which is why we are not expecting this to be a major issue for our analyses [34].

The pandemic phases used to determine the interruption points and defined by Tolksdorf et al. [20] are based on different parameters like notified cases of COVID-19, number of tests, hospitalisation incidence, and intensive care capacities. The public health and social measures in Germany were largely also driven by those parameters, but were, apart from the first “lockdown” in March 2020, not uniform across different German states. This might affect the interpretability of the present results concerning the impact of public health and social measures. Additionally, there is no substantial evidence to back up assumptions about delay periods between the introduction of specific public health and social measures and healthcare utilisation. In the present study, the two-week delay period was therefore chosen based on the best model fit. The pandemic phases include different seasons. However, we were able to include data since March 2017, allowing us to use three pre-pandemic years as a reference period, where there was no substantial seasonal variation visible.

## Conclusions

Analysing data from a syndromic surveillance system using emergency department data can retrospectively contribute to the wider understanding of the impact of the COVID-19 pandemic and associated public health and social measures. However, given the implemented surveillance system at RKI where data is available on a daily basis, changes in health-care seeking behaviour could also be detected instantaneously and be routinely fed into public health decision-making. The basis of both of these aims is the analysis of changes in consultation numbers and the distribution of patient characteristics, as presented in this study.

The presented results show that while the overall number of consultations decreased during each phase of the COVID-19 pandemic compared to the time before the pandemic, the distribution of patient characteristics did not vary extensively. Especially older age groups and those presenting with more severe complaints showed the smallest relative decrease in each pandemic phase. This indicates that patients assumed to be more vulnerable did receive necessary emergency treatment and were not lacking access to emergency departments.

Better and early understanding of the population’s reasons for emergency care avoidance can in the future help target up-to-date public health messaging and the development of strategies to increase confidence in the safety of healthcare institutions despite pandemic situations.

## Supplementary Information

**Additional file 1: Supplementary Fig. 1.** Model fit for overall consultations showing number of cases per week (black) and fitted values (red) for A): a two-week delay, B): a one-week delay and C): no delay**. Supplementary Fig. 2.** Relative number of emergency department consultations stratified by weekday of consultation comparing the pandemic phases**. Supplementary Fig. 3.** Relative number of emergency department consultations stratified by hour of consultation comparing the pandemic phases**. Supplementary Fig. 4.** Relative change in % and 95 confidence interval for all emergency department consultations, comparing every pandemic phase with the pre-pandemic reference period**. Supplementary Fig. 5.** Relative change in % and 95 confidence interval for all emergency department consultations by weekday, comparing every pandemic phase with the pre-pandemic reference period**. Supplementary Fig. 6.** Relative change in % and 95 confidence interval for all emergency department consultations by hour of day, comparing every pandemic phase with the pre-pandemic reference period**. Supplementary Table 1.** Relative percentage change, 95% confidence interval and *p*-value for the pre-pandemic trend (i.e. linear time from start of study period until start of first interruption)**. Supplementary Table 2.** Absolute and relative number of all emergency department consultations, consultations by age group, acuity level, weekday, and hour of day, stratified by pandemic phases.

## Data Availability

The data used and analysed during the current study are available on reasonable request upon contacting the corresponding author (schranzm@rki.de).

## Abbreviations

AIC: Akaike information criterion
AKTIN: Aktionsbündnis zur Verbesserung der Kommunikations- und InformationsTechnik in der Intensiv- und Notfallmedizin (AKTIN emergency department data registry)
CI: Confidence Interval
COVID-19: Coronavirus Disease 2019
ED: Emergency department
ESEG: Erkennung und Sicherung epidemischer Gefahrenlagen
NoKeDa: NotaufnahmeKernDatenmodell
RKI: Robert Koch Institute
SARS-CoV-2: Severe acute respiratory syndrome coronavirus 2
UK: United Kingdom
US: United States
