# Supplementary material for: Changes in emergency department utilisation in Germany before and during different phases of the COVID-19 pandemic, using data from a national surveillance system up to June 2021
Source: BMC Public Health. 2023 May 2;23:799. doi: 10.1186/s12889-023-15375-7 (PMC10152015; doi:10.1186/s12889-023-15375-7)
Supplement: Supplementary file 1 — Additional file 1: Supplementary Fig. 1. Model fit for overall consultations showing number of cases per week (black) and fitted values (red) for A): a two-week delay, B): a one-week delay and C): no delay. Supplementary Fig. 2. Relative number of emergency department consultations stratified by weekday of consultation comparing the pandemic phases. Supplementary Fig. 3. Relative number of emergency department consultations stratified by hour of consultation comparing the pandemic phases. Supplementary Fig. 4. Relative change in % and 95 confidence interval for all emergency department consultations, comparing every pandemic phase with the pre-pandemic reference period. Supplementary Fig. 5. Relative change in % and 95 confidence interval for all emergency department consultations by weekday, comparing every pandemic phase with the pre-pandemic reference period. Supplementary Fig. 6. Relative change in % and 95 confidence interval for all emergency department consultations by hour of day, comparing every pandemic phase with the pre-pandemic reference period. Supplementary Table 1. Relative percentage change, 95% confidence interval and p-value for the pre-pandemic trend (i.e. linear time from start of study period until start of first interruption). Supplementary Table 2. Absolute and relative number of all emergency department consultations, consultations by age group, acuity level, weekday, and hour of day, stratified by pandemic phases. [file 12889_2023_15375_MOESM1_ESM.docx]

#
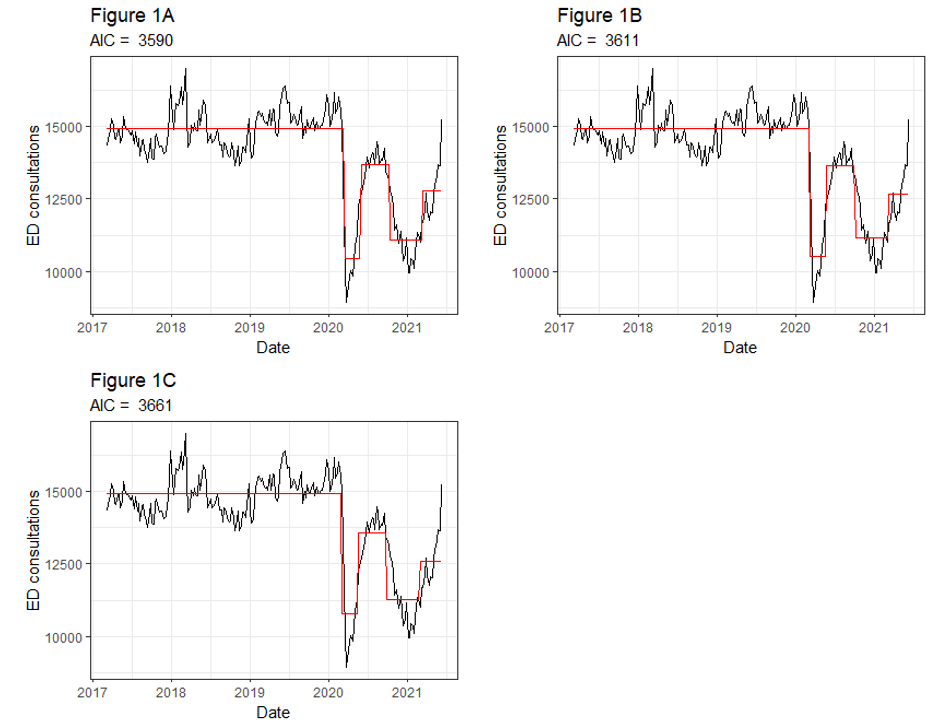
Supplementary Figures

Supplementary Figure 1 - Model fit for overall consultations showing number of cases per week (black) and fitted values (red) for A): a two-week delay, B): a one-week delay and C): no delay

|  |  |
| --- | --- |
|  |  |

**
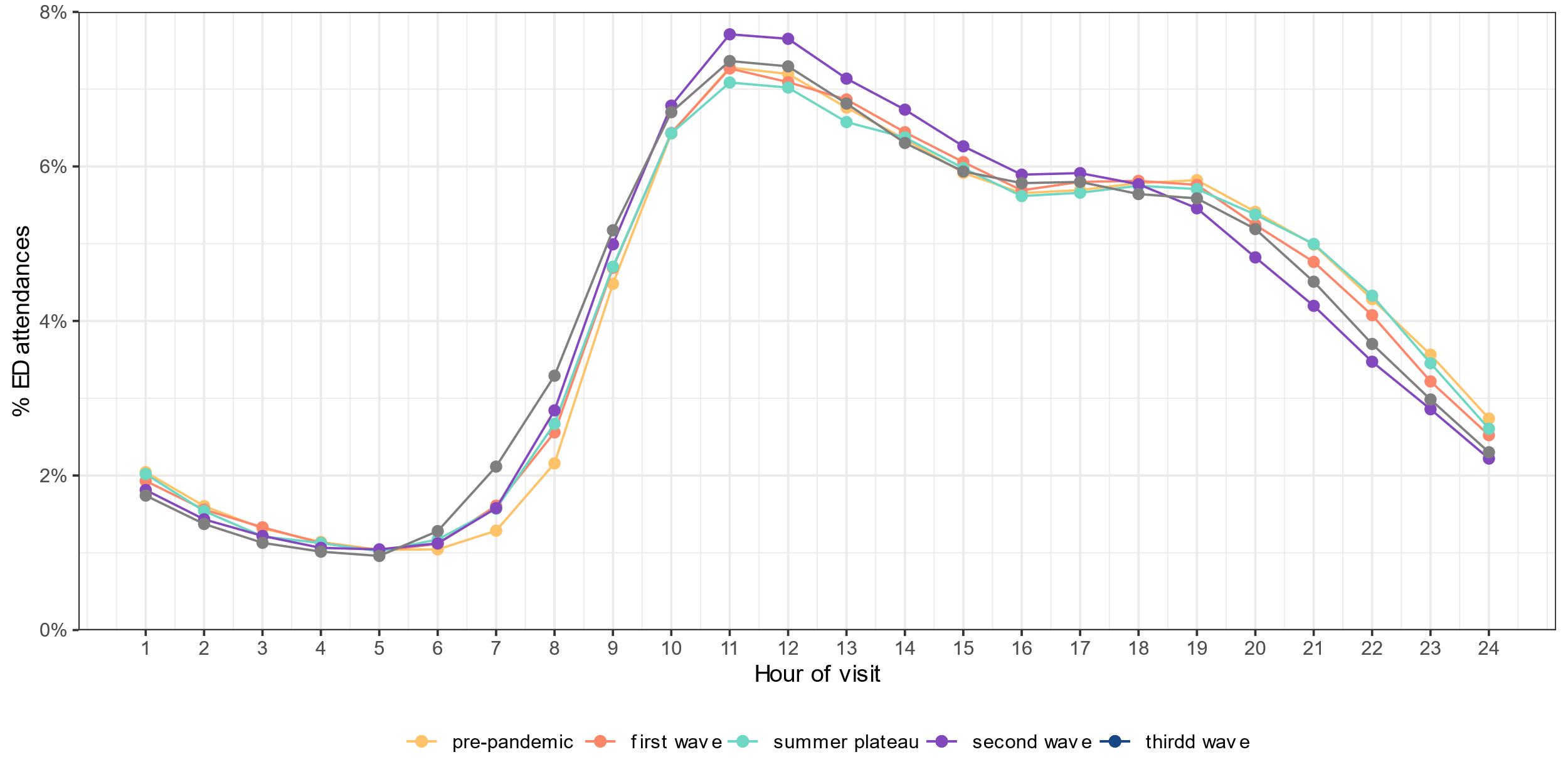

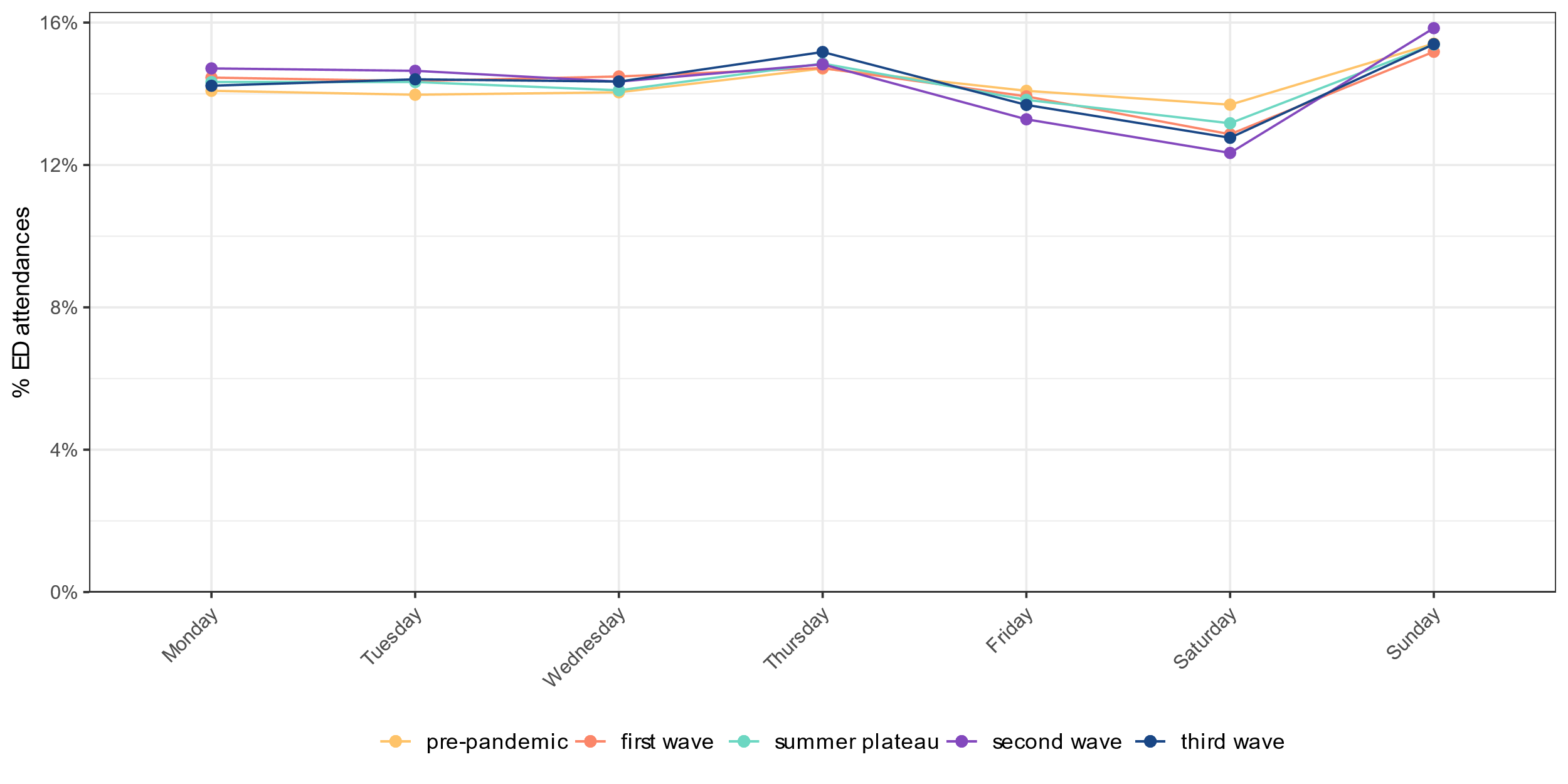
**

Supplementary Figure 3 Relative number of emergency department consultations stratified by hour of consultation comparing the pandemic phases

Supplementary Figure 2 - Relative number of emergency department consultations stratified by weekday of consultation comparing the pandemic phases

**
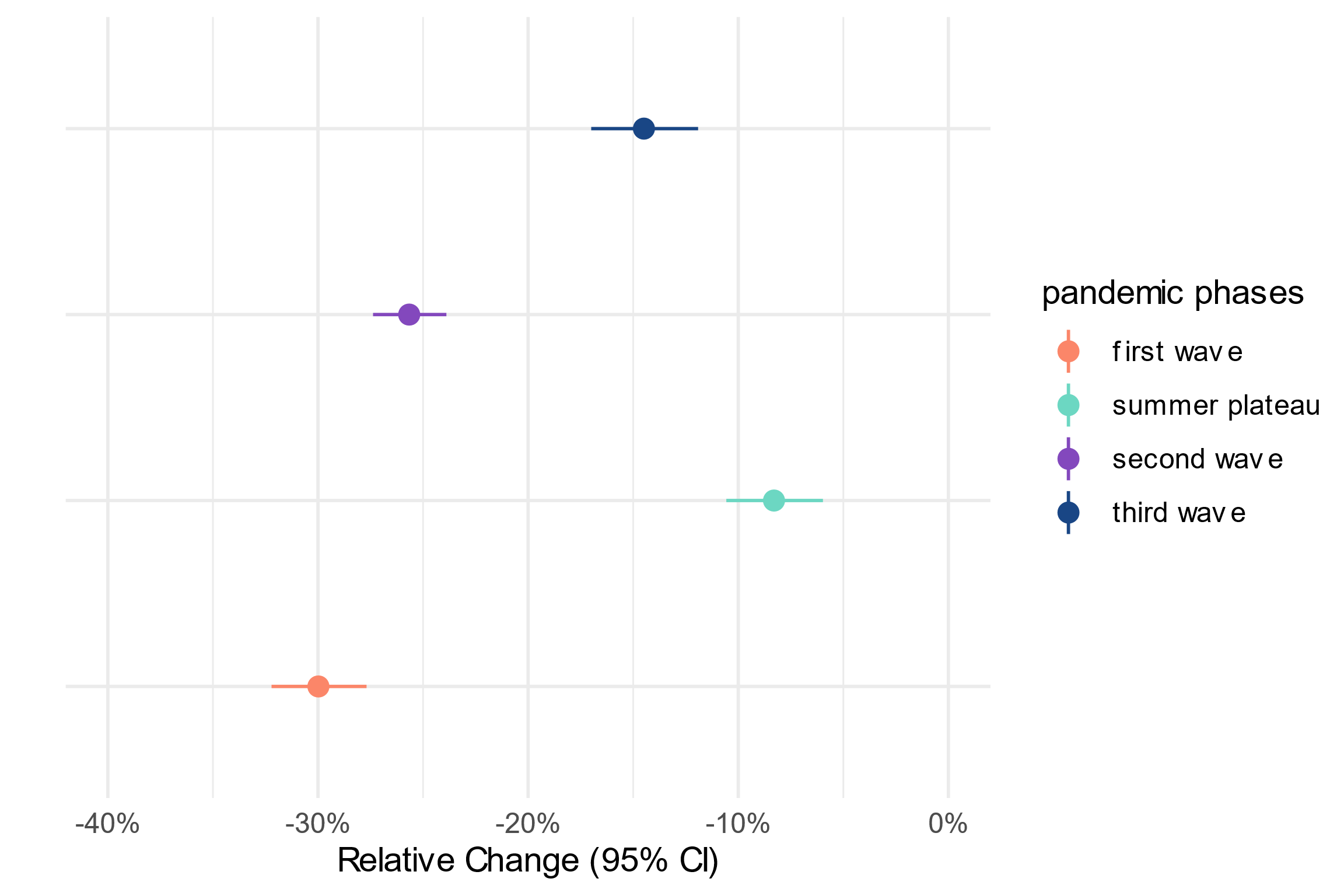
**

Supplementary Figure 4 - Relative change in % and 95% confidence interval for all emergency department consultations, comparing every pandemic phase with the pre-pandemic reference period


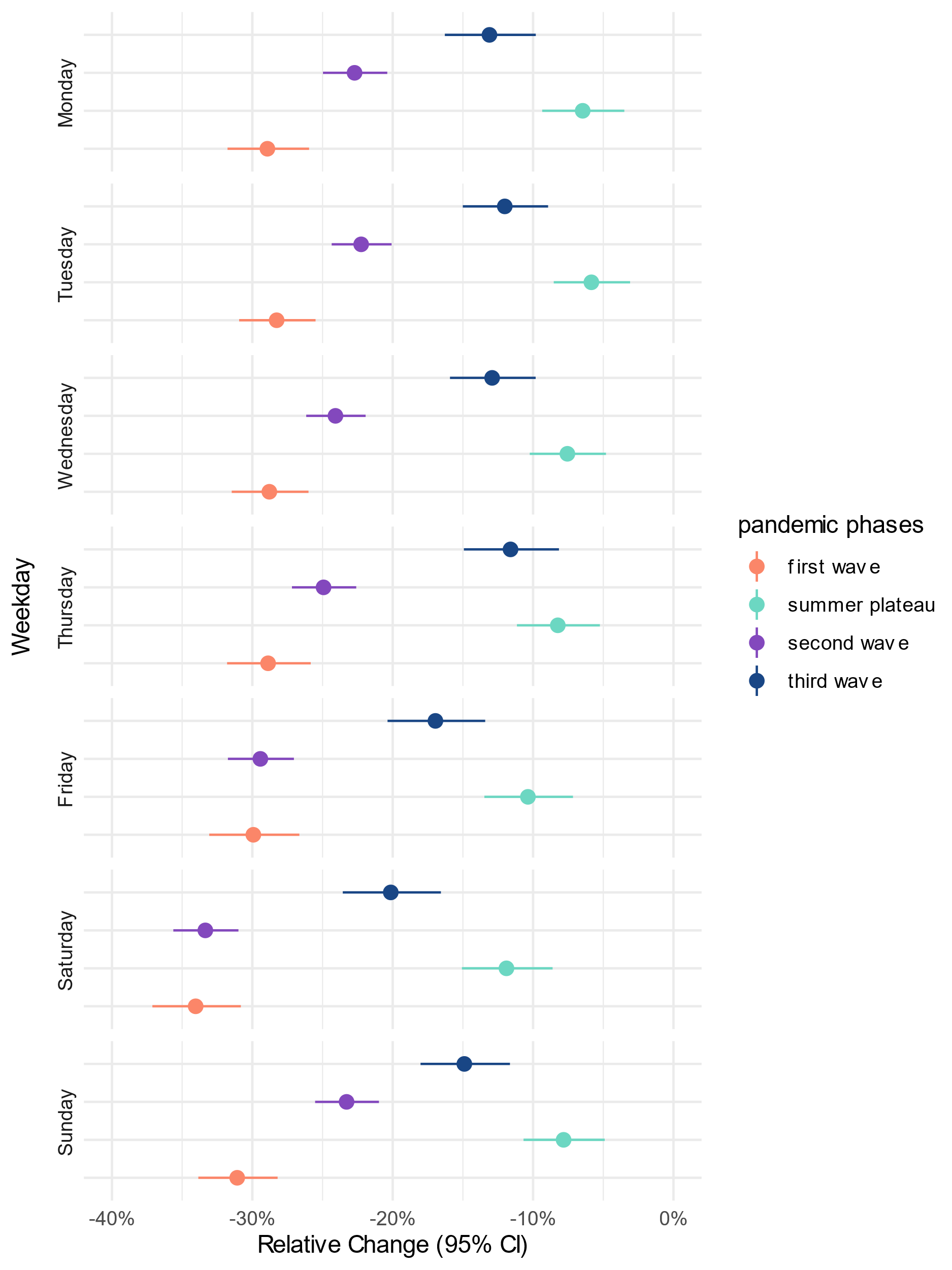


Supplementary Figure 5 - Relative change in % and 95% confidence interval for all emergency department consultations by weekday, comparing every pandemic phase with the pre-pandemic reference period


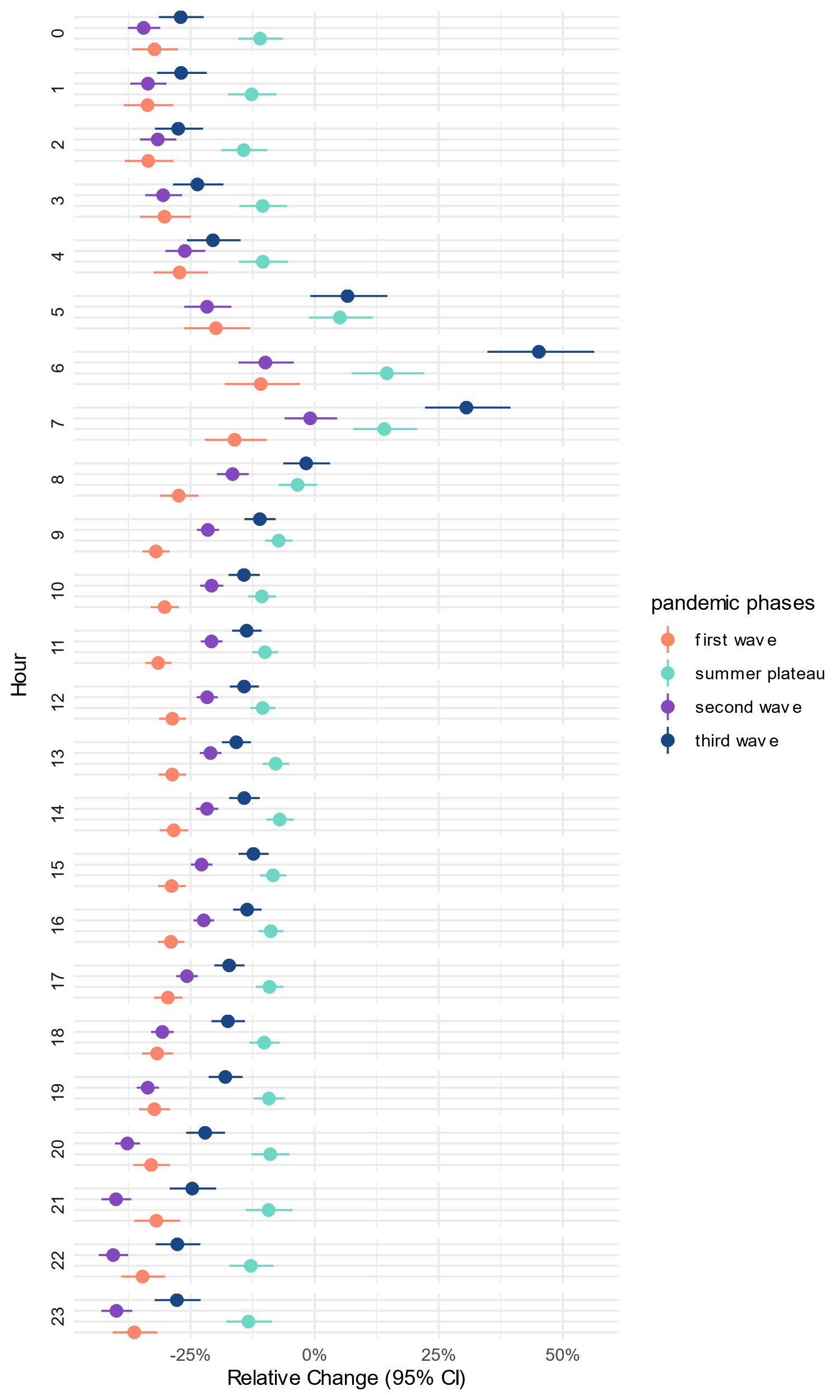


Supplementary Figure 6 - Relative change in % and 95% confidence interval for all emergency department consultations by hour of day, comparing every pandemic phase with the pre-pandemic reference period

# Supplementary Tables

Supplementary Table 1 - Relative percentage change, 95% confidence interval and p-value for the pre-pandemic trend (i.e. linear time from start of study period until start of first interruption)

|  | **% Change** | **2.5%** | **97.5%** |
| --- | --- | --- | --- |
| **All consultations** |  |  |  |
|  | 0.03 | 0.01 | 0.05 |
| **Age group** |  |  |  |
| 0-19 | 0.04 | 0.03 | 0.04 |
| 20-39 | -0.01 | -0.01 | -0.00 |
| 40-59 | -0.00 | -0.00 | 0.00 |
| 60-79 | 0.01 | 0.00 | 0.01 |
| 80+ | -0.00 | -0.01 | -0.00 |
| **Acuity level** |  |  |  |
| 1 (immediate) | -0.01 | -0.02 | -0.00 |
| 2 (very urgent) | 0.00 | -0.00 | 0.01 |
| 3 (urgent) | 0.01 | 0.01 | 0.01 |
| 4 (standard) | 0.00 | -0.00 | 0.01 |
| 5 (non urgent) | 0.02 | 0.01 | 0.02 |
| Missing | -0.01 | -0.02 | -0.01 |
| **Weekday** |  |  |  |
| Monday | 0.01 | 0.00 | 0.01 |
| Tuesday | 0.01 | 0.00 | 0.01 |
| Wednesday | 0.01 | 0.00 | 0.01 |
| Thursday | 0.01 | 0.00 | 0.01 |
| Friday | -0.00 | -0.00 | 0.00 |
| Saturday | -0.00 | -0.00 | 0.00 |
| Sunday | 0.01 | 0.00 | 0.01 |
| **Hour** |  |  |  |
| 0 | 0.00 | 0.00 | 0.00 |
| 1 | 0.00 | 0.00 | 0.01 |
| 2 | 0.00 | 0.00 | 0.00 |
| 3 | 0.00 | 0.00 | 0.00 |
| 4 | 0.00 | 0.00 | 0.01 |
| 5 | 0.00 | 0.00 | 0.01 |
| 6 | 0.01 | 0.00 | 0.01 |
| 7 | 0.01 | 0.01 | 0.01 |
| 8 | 0.00 | 0.00 | 0.00 |
| 9 | 0.00 | 0.00 | 0.00 |
| 10 | 0.00 | 0.00 | 0.00 |
| 11 | 0.00 | 0.00 | 0.00 |
| 12 | 0.00 | 0.00 | 0.00 |
| 13 | 0.00 | 0.00 | 0.00 |
| 14 | 0.00 | 0.00 | 0.00 |
| 15 | 0.00 | 0.00 | 0.00 |
| 16 | 0.00 | 0.00 | 0.00 |
| 17 | 0.00 | -0.00 | 0.00 |
| 18 | -0.00 | -0.00 | 0.00 |
| 19 | -0.00 | -0.00 | 0.00 |
| 20 | -0.00 | -0.00 | 0.00 |
| 21 | -0.00 | -0.00 | 0.00 |
| 22 | -0.00 | -0.00 | 0.00 |
| 23 | 0.00 | -0.00 | 0.00 |

Supplementary Table 2 - Absolute and relative number of all emergency department consultations, consultations by age group, acuity level, weekday, and hour of day, stratified by pandemic phases

|  | *N (%)* | | | | | |
| --- | --- | --- | --- | --- | --- | --- |
|  | **Pre-Pandemic** | **Wave 1** | **Summerbreak** | **Wave 2** | **Wave 3** |  |
| Weeks per phase | 156 | 19 | 11 | 22 | 15 |  |
| Ø attendances per week | 14,935.53 | 13,586.26 | 10,783.55 | 11,271.64 | 12,573.07 |  |
| **All attendances** |  |  |  |  |  |  |
|  | 2,329,943 (100%) | 258,139 (100%) | 118,619 (100%) | 247,976 (100%) | 188,596 (100%) |  |
| **Age group** |  |  |  |  |  |  |
| 0-19 | 432,090 (18.5%) | 47,879 (18.5%) | 20,420 (17.2%) | 41,000 (16.5%) | 33,995 (18.0%) |  |
| 20-39 | 530,985 (22.8%) | 56,769 (22.0%) | 25,904 (21.8%) | 52,101 (21.0%) | 40,562 (21.5%) |  |
| 40-59 | 480,819 (20.6%) | 54,098 (21.0%) | 25,291 (21.3%) | 51,765 (20.9%) | 39,464 (20.9%) |  |
| 60-79 | 514,119 (22.1%) | 59,083 (22.9%) | 27,812 (23.4%) | 59,570 (24.0%) | 44,104 (23.4%) |  |
| 80+ | 371,930 (16.0%) | 40,310 (15.6%) | 19,192 (16.2%) | 43,540 (17.6%) | 30,471 (16.2%) |  |
| **Severity level** |  |  |  |  |  |  |
| 1 (immediate) | 26,624 (1.1%) | 3,048 (1.2%) | 1,458 (1.2%) | 3,366 (1.4%) | 2,503 (1.3%) |  |
| 2 (very urgent) | 267,807 (11.5%) | 30,095 (11.7%) | 14,551 (12.3%) | 32,975 (13.3%) | 23,437 (12.4%) |  |
| 3 (urgent) | 866,551 (37.2%) | 100,352 (38.9%) | 46,105 (38.9%) | 100,713 (40.6%) | 76,055 (40.3%) |  |
| 4 (standard) | 944,203 (40.5%) | 100,611 (39.0%) | 43,345 (36.5%) | 87,896 (35.4%) | 69,972 (37.1%) |  |
| 5 (non urgent) | 92,667 (4.0%) | 10,937 (4.2%) | 4,617 (3.9%) | 9,439 (3.8%) | 7,199 (3.8%) |  |
| Missing | 132,091 (5.7%) | 13,096 (5.1%) | 8,543 (7.2%) | 13,587 (5.5%) | 9,430 (5.0%) |  |
| **Weekday** |  |  |  |  |  |  |
| Monday | 328,166 (14.1%) | 36,994 (14.3%) | 17,148 (14.5%) | 36,487 (14.7%) | 26,832 (14.2%) |  |
| Tuesday | 325,575 (14.0%) | 36,997 (14.3%) | 17,031 (14.4%) | 36,319 (14.6%) | 27,172 (14.4%) |  |
| Wednesday | 327,168 (14.0%) | 36,396 (14.1%) | 17,185 (14.5%) | 35,567 (14.3%) | 27,052 (14.3%) |  |
| Thursday | 342,753 (14.7%) | 38,335 (14.9%) | 17,466 (14.7%) | 36,771 (14.8%) | 28,619 (15.2%) |  |
| Friday | 328,159 (14.1%) | 35,700 (13.8%) | 16,521 (13.9%) | 32,941 (13.3%) | 25,815 (13.7%) |  |
| Saturday | 319,062 (13.7%) | 34,004 (13.2%) | 15,259 (12.9%) | 30,599 (12.3%) | 24,073 (12.8%) |  |
| Sunday | 359,060 (15.4%) | 39,713 (15.4%) | 18,009 (15.2%) | 39,292 (15.8%) | 29,033 (15.4%) |  |
| **Hour** |  |  |  |  |  |  |
| 0 | 47,639 (2.0%) | 5,214 (2.0%) | 2,289 (1.9%) | 4,495 (1.8%) | 3,283 (1.7%) |  |
| 1 | 37,369 (1.6%) | 3,977 (1.5%) | 1,849 (1.6%) | 3,553 (1.4%) | 2,589 (1.4%) |  |
| 2 | 30,665 (1.3%) | 3,131 (1.2%) | 1,578 (1.3%) | 3,021 (1.2%) | 2,130 (1.1%) |  |
| 3 | 26,565 (1.1%) | 2,908 (1.1%) | 1,338 (1.1%) | 2,638 (1.1%) | 1,913 (1.0%) |  |
| 4 | 24,219 (1.0%) | 2,631 (1.0%) | 1,216 (1.0%) | 2,590 (1.0%) | 1,809 (1.0%) |  |
| 5 | 24,323 (1.0%) | 3,028 (1.2%) | 1,324 (1.1%) | 2,783 (1.1%) | 2,416 (1.3%) |  |
| 6 | 29,963 (1.3%) | 4,056 (1.6%) | 1,909 (1.6%) | 3,913 (1.6%) | 3,991 (2.1%) |  |
| 7 | 50,278 (2.2%) | 6,892 (2.7%) | 3,035 (2.6%) | 7,050 (2.8%) | 6,208 (3.3%) |  |
| 8 | 104,405 (4.5%) | 12,140 (4.7%) | 5,562 (4.7%) | 12,381 (5.0%) | 9,759 (5.2%) |  |
| 9 | 149,740 (6.4%) | 16,597 (6.4%) | 7,632 (6.4%) | 16,832 (6.8%) | 12,641 (6.7%) |  |
| 10 | 169,646 (7.3%) | 18,294 (7.1%) | 8,622 (7.3%) | 19,121 (7.7%) | 13,890 (7.4%) |  |
| 11 | 167,706 (7.2%) | 18,124 (7.0%) | 8,412 (7.1%) | 18,976 (7.7%) | 13,760 (7.3%) |  |
| 12 | 157,565 (6.8%) | 16,972 (6.6%) | 8,146 (6.9%) | 17,699 (7.1%) | 12,852 (6.8%) |  |
| 13 | 148,070 (6.4%) | 16,459 (6.4%) | 7,643 (6.4%) | 16,702 (6.7%) | 11,890 (6.3%) |  |
| 14 | 137,867 (5.9%) | 15,431 (6.0%) | 7,185 (6.1%) | 15,526 (6.3%) | 11,194 (5.9%) |  |
| 15 | 131,759 (5.7%) | 14,500 (5.6%) | 6,751 (5.7%) | 14,616 (5.9%) | 10,909 (5.8%) |  |
| 16 | 132,678 (5.7%) | 14,611 (5.7%) | 6,881 (5.8%) | 14,666 (5.9%) | 10,939 (5.8%) |  |
| 17 | 134,792 (5.8%) | 14,843 (5.8%) | 6,896 (5.8%) | 14,308 (5.8%) | 10,643 (5.6%) |  |
| 18 | 135,677 (5.8%) | 14,734 (5.7%) | 6,835 (5.8%) | 13,536 (5.5%) | 10,537 (5.6%) |  |
| 19 | 126,143 (5.4%) | 13,883 (5.4%) | 6,221 (5.2%) | 11,961 (4.8%) | 9,789 (5.2%) |  |
| 20 | 116,228 (5.0%) | 12,899 (5.0%) | 5,652 (4.8%) | 10,406 (4.2%) | 8,503 (4.5%) |  |
| 21 | 99,800 (4.3%) | 11,172 (4.3%) | 4,834 (4.1%) | 8,612 (3.5%) | 6,983 (3.7%) |  |
| 22 | 83,061 (3.6%) | 8,916 (3.5%) | 3,817 (3.2%) | 7,088 (2.9%) | 5,628 (3.0%) |  |
| 23 | 63,785 (2.7%) | 6,727 (2.6%) | 2,992 (2.5%) | 5,503 (2.2%) | 4,340 (2.3%) |  |
